# 2186 Feasibility of maternal holding during therapeutic hypothermia for infants with encephalopathy

**DOI:** 10.1017/cts.2018.168

**Published:** 2018-11-21

**Authors:** Alexa Kanwit Craig, Kyle Deerwester, Leah Fox, Julia Jacobs, Scott Evans

**Affiliations:** 1 Tufts University; 2 Tufts University School of Medicine; 3 Maine Medical Center; 4 Walter Reed National Military Medical Center; 5 Maine Medical Center

## Abstract

OBJECTIVES/SPECIFIC AIMS: Therapeutic hypothermia (TH) is a neuroprotective therapy regularly used in newborn infants following traumatic births. The infant’s temperature is maintained at 33.5°C for 72 hours by a cooling blanket upon which the infant is placed. Parents are not permitted to hold their infant while TH is ongoing due to concerns for unintentional rewarming or accidental dislodging of catheters or other monitoring equipment. Our prior qualitative research with nurse and parent interviews described the inability to hold an infant during TH as a significant source of stress. We assessed the feasibility of a 30-minute period of maternal holding for infants being actively treated with TH and assessed both the maternal experience of holding and the nurse experience of supporting holding. METHODS/STUDY POPULATION: This was a feasibility study employing a mixed-methods approach. Inclusion criteria were gestational age at birth of 35 weeks or greater, absence of clinical or electrographic seizures during the first 24 hours of TH, and designation as “clinically stable” by the attending neonatologist with the infant on room air, nasal cannula, or continuous positive airway pressure. Quantitative data were obtained from vital sign monitoring every 2 minutes before, during and after holding and from maternal and nurse research surveys. Qualitative data were obtained from nurse surveys. Infant rewarming was prevented through use of a thin foam insulating barrier placed between mother and infant during holding. Adverse events were defined as a change in infant temperature greater than 0.5°C above or below 33.5°C, accidental dislodging of central lines/disruption of EEG leads or early termination of holding due vital sign instability present for greater than 2 recorded measurements including infant bradycardia defined as heart rate less than 80 beats per minute, hypotension defined as mean arterial pressure less than 40 mmHg or oxygen saturation of less than 93%. RESULTS/ANTICIPATED RESULTS: There were 10 newborn infants undergoing TH for neonatal encephalopathy (median gestational age 39.4 weeks) and their mothers (median age=31 years) were recruited. Infants remained on the hypothermia blanket during holding and were transferred safely to their mother’s arms without medical equipment malfunction/dislodgement. Holding occurred at a median of 47 hours of life. The mean temperature prior to holding was 33.4°C and at completion of holding the mean temperature was 33.5°C (*p*=0.18). There were no significant bradycardia, hypotension or oxygen desaturation events. In total, 80% of mothers reported difficulty bonding with their baby prior to holding and 90% reported a high level of stress before holding. After holding, all mothers felt their bond was “stronger” or “much stronger” and all felt “less stressed” or “much less stressed.” After holding, 75% of nurses reported that they felt a more positive emotional response to the infant. One nurse stated, “being a part of this emotional experience made me feel closer and more connected to this family and gave me a different perspective on just what they had been dealing with and feeling since giving birth to their child.” In free text responses, on 5 separate occasions, nurses commented on the relaxed, calmed or less irritable appearance of the infant while being held during TH. DISCUSSION/SIGNIFICANCE OF IMPACT: In this sample of term infants treated with TH, a 30-minute period of maternal holding was not associated with increased temperature or other adverse events. Holding during TH was associated with extremely positive feedback from mothers and nurses. Future larger studies could consider assessing the impact of holding on endocrinological markers of stress and bonding, on infant glycemic control, on breastfeeding success rates, and the impact of earlier and improved bonding on the developmental outcomes of children held during their treatment with TH. Increasing the duration of holding and allowing both parents to hold on more than one occasion during the 72 hours of TH may increase the proposed benefits of this intervention.

